# Effects of Solvent Diols on the Synthesis of ZnFe_2_O_4_ Particles and Their Use as Heterogeneous Photo-Fenton Catalysts

**DOI:** 10.3390/ma7096281

**Published:** 2014-09-03

**Authors:** Chayene Gonçalves Anchieta, Adriano Cancelier, Marcio Antonio Mazutti, Sérgio Luiz Jahn, Raquel Cristine Kuhn, Andre Gündel, Osvaldo Chiavone-Filho, Edson Luiz Foletto

**Affiliations:** 1 Department of Chemical Engineering, Federal University of Santa Maria, 97105-900 Santa Maria, Brazil; E-Mails: chayeneanchieta@gmail.com (C.G.A.); adriano.cancelier@ufsm.br (A.C.); mazutti@ufsm.br (M.A.M.); jahn@smail.ufsm.br (S.L.J.); raquel.kuhn@ufsm.br (R.C.K.); 2 Department of Physics, Federal University of Pampa, 96413-170 Bagé, Brazil; E-Mail: andre.gundel@unipampa.edu.br; 3 Department of Chemical Engineering, Federal University of Rio Grande do Norte, 59066-800 Natal, Brazil; E-Mail: osvaldo@eq.ufrn.br

**Keywords:** zinc ferrite, ZnFe_2_O_4_, synthesis, solvothermal, photo-Fenton

## Abstract

A solvothermal method was used to prepare zinc ferrite spinel oxide (ZnFe_2_O_4_) using ethylene glycol and 1,4 butanediol as solvent diols, and the influence of diols on the physical properties of ZnFe_2_O_4_ particles was investigated. The produced particles were characterized by X-ray powder diffraction (XRD), atomic force microscopy (AFM), Fourier transform infrared spectroscopy (FTIR) and nitrogen adsorption isotherms, and the catalytic activity for the organic pollutant decomposition by heterogeneous photo-Fenton reaction was investigated. Both solvents produced particles with cubic spinel structure. Microporous and mesoporous structures were obtained when ethylene glycol and 1,4 butanediol were used as diols, respectively. A higher pore volume and surface area, as well as a higher catalytic activity for the pollutant degradation were found when 1,4 butanediol was used as solvent.

## 1. Introduction

Zinc ferrite (ZnFe_2_O_4_) is a spinel oxide that possesses excellent magnetic and electrical properties [1,2], as well as excellent chemical and thermal stabilities [3]. ZnFe_2_O_4_ oxide has received much attention due to its potential applications in detecting gases [4], as an adsorbent material for hot-gas desulfurization [5], in biomedicine [6], for its magnetic, optical and electrical behaviors [7,8,9,10,11] and catalytic application [12,13]. Recently, zinc ferrite has been used as an efficient heterogeneous Fenton catalyst in degrading organic pollutants from an aqueous solution [14,15,16]. ZnFe_2_O_4_ nanoparticles were developed as a catalyst for the degradation of benzotriazole by a heterogeneous photoelectron-Fenton process and have shown to be highly efficient for benzotriazole degradation [16]. A hydrothermal method was used to synthesize ZnFe_2_O_4_ powders with an average size of 10 nm with the aid of sodium oleate, and they presented good photocatalytic activity in the degradation of Rhodamine B dye under the irradiation of simulated solar light [17]. ZnFe_2_O_4_ film fabricated on a sulfonated silicon substrate via a novel template-assisted route exhibited good photocatalytic activity in the degradation of Rhodamine B under visible light irradiation [18]. ZnFe_2_O_4_ nanocrystallites were synthesized by microwave sintering and played an important role in degrading the methylene blue dye under visible light [19].

ZnFe_2_O_4_ particles have been prepared using various methods, such as co-precipitation [20,21], sol-gel [22], solid-state reaction [23], glycine combustion method [24], combustion reaction using urea as reducing agent [25,26], hydrothermal synthesis [27], solvothermal and microwave-assisted solvothermal synthesis [28], high enegy ball-milling [29], thermal plasma synthesis [30], one-step solid-phase chemical reaction [31], microwave combustion method [32], polyethylene glycol-assisted route [33] and synthesis in supercritical fluids [34,35]. Herein, we report the use of a solvothermal route for the preparation of ZnFe_2_O_4_ particles. A solvothermal route offers advantage over the hydrothermal route, because it does not require the use of surfactants or templates in the reaction medium. The solvothermal method was used to fabricate ZnFe_2_O_4_/α-Fe_2_O_3_ composite hollow nanospheres, including polyethylene glycol as template [36]. Li *et al.* [37] and Kuai *et al.* [38] used ethylene glycol as solvent for the synthesis of ZnFe_2_O_4_ nanospheres and Ce^3+^ doped Zn ferrites, respectively.

Accordingly, this work aimed to synthesize ZnFe_2_O_4_ powders with a solvothermal route, using different solvent diols, and to examine their structural properties. In addition, the catalytic performance for organic dye degradation over ZnFe_2_O_4_ powders was investigated.

## 2. Experimental Section

### 2.1. Preparation of Powders

The ZnFe_2_O_4_ particles were prepared using the solvothermal method. Zinc nitrate (Zn(NO_3_)_2_·6H_2_O, analytical grade) and iron nitrate (Fe(NO_3_)_3_·9H_2_O, analytical grade) were used as zinc and iron sources, respectively, without further purification. Stoichiometric amounts of Zn and Fe nitrates (molar ratio Zn:Fe = 1:2) were used for preparing ZnFe_2_O_4_ powders. Two diols were used was solvent, ethylene glycol (C_2_H_4_(OH)_2_, analytical grade) and 1,4 butanediol (C_4_H_10_O_2_, analytical grade). In a typical synthetic procedure, zinc nitrate (4 mmol) and iron nitrate (8 mmol) were dissolved in 120 mL of ethylene glycol (EG) and mixed with appropriate amount of sodium acetate (CH_3_COONa) (60 mmol), under magnetic stirring. Then, the final mixture was charged into a PTFE-lined stainless autoclave, and the solvothermal reaction was carried out at 200 °C for 24 h. Subsequently, the autoclave was left to naturally cool off. The solids were filtered, washed with distilled water, and dried at 110 °C for 10 h to obtain ZnFe_2_O_4_-EG. A similar procedure to that described above was carried out using 1,4 butanediol (BD) to obtain ZnFe_2_O_4_-DB.

### 2.2. Characterization of Powders

The XRD patterns were obtained on a Rigaku Miniflex 300 diffractometer with a Cu *K*α radiation at 30 kV and 10 mA, with a step size (2θ) of 0.03° and a count time of 0.9 s per step. The average size of the ZnFe_2_O_4_ spinel crystallite was determined with the Scherrer equation [39]: *D* = *K*·λ/(*h*_1/2_·cosθ), where *D* is the average crystallite size, *K* the Scherrer constant (0.9), λ the wavelength of incident X-rays (0.1541 nm), *h*_1/2_ the peak width at half height and θ corresponds to the peak position (in this work, 2θ = 35.36°). The AFM images were obtained by atomic force microscopy (Agilent Technologies 5500 equipment). N_2_ adsorption-desorption isotherms measurements were carried out at 77 K using an ASAP 2020 apparatus, at a relative pressure (*P*/*P*_0_) from 0 to 0.99. FTIR spectra were recorded on a Shimadzu IR-Prestige-21 spectrophotometer in the range of 4000–375 cm^−1^, using pellets prepared by mixing zinc ferrite powder with KBr powder (10 mg zinc ferrite/300 mg KBr).

### 2.3. Experimental Essays and Reaction Apparatus

A batch–type reactor was used, consisting of a glass tube (internal diameter of 5.0 cm and 6.0 cm in height) with an economic fluorescent lamp (80 W, emit at wavelength above 400 nm) fixed above the reaction solution. Due to the narrow bandgap of ~1.9 eV [37,40], ZnFe_2_O_4_ shows a wide absorption in the visible-light region and could be easily excited by visible light, accelerating the degradation of organic molecules from an aqueous solution. Visible light assisted Fenton system for the treatment of dyes has been shown to be very promising [41,42]. The reaction solution was 15 cm apart from the lamp. For the catalytic experiments under visible irradiation, 0.5 g of catalyst was added to 50 mL of Procion Red dye aqueous solution at an initial concentration of 50 mg·L^−1^, followed by adjusting pH to 3.0 by 0.1 M H_2_SO_4_. Acidic conditions (about pH 3) are required for a better performance of Fenton reaction [41,43]. Previous to irradiation, the suspension was magnetically stirred in the dark until reaching the adsorption equilibrium. After the adsorption process, an aliquot of hydrogen peroxide (0.04 mol·L^−1^) was added to the solution to initiate the reaction. When H_2_O_2_ was added, it greatly enhanced the efficiency of degradation, which affects –OH production for the rapid oxidation of contaminants [42,43]. Then the suspension was irradiated by the lamp, and aliquots were collected at set time intervals using a 5 mL syringe, followed by the filtration of the suspension. The reaction was always kept at room temperature. Dye concentration data were treated in the dimensionless form (*C/C*_0_ = *A/A*_0_) and plotted as a function of reaction time, where *C*_0_ represents the absorbance of the initial dye solution and *C* the absorbance of the dye solution at reaction time *t*. The absorbance was measured using a UV-Vis spectrophotometer (Bel Photonics, SP1105, Bel Photonics do Brasil Ltda., Osasco, Brazil) at maximum wavelength of 543 nm. The concentration of Fe irons leaching from ZnFe_2_O_4_ particles during the reaction process was measured using atomic absorption spectroscopy (Agilent Technologies, 200 series AA (Agilent Technologies, Inc., Santa Clara, CA, USA).

## 3. Results and Discussion

**Figure 1:**
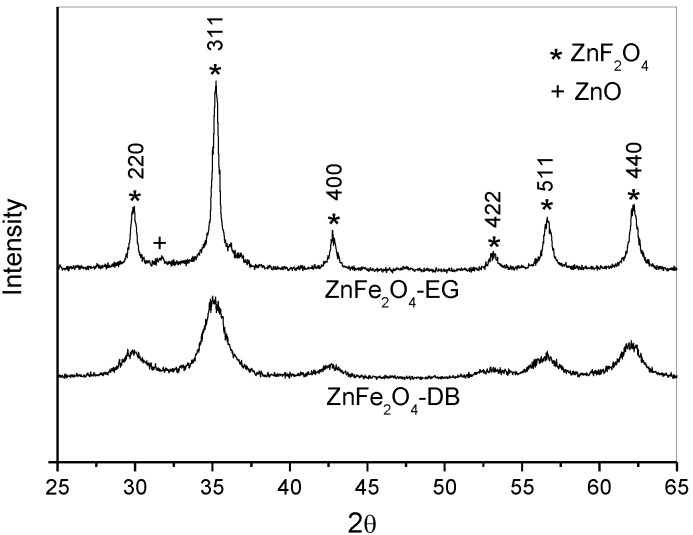
XRD patterns of the samples prepared with different solvent diols.

Figure 1 shows the XRD patterns of ZnFe_2_O_4_ samples prepared with EG and BD. The diffractograms for both samples indicate that each sample corresponds to a spinel cubic structure according to JCPDS card No. 89-1012. The diffraction peaks at 2θ of 30.05°, 35.36°, 42.78°, 52.96°, 56.78° and 62.2° can be ascribed to the reflection of (220), (311), (400), (422), (511) and (440) planes of the ZnFe_2_O_4_ spinel, respectively. However, a very small amount of ZnO (2θ = 31.7°) was detected in ZnFe_2_O_4_ synthesized with ethylene glycol, as shown in Figure 1. The main difference in the X-ray diffractograms of the ZnFe_2_O_4_ samples prepared with EG and BD is the width of the peaks. It may be noted that the ZnFe_2_O_4_-BD sample has wider peaks than those of ZnFe_2_O_4_-EG. This indicates that the ZnFe_2_O_4_-BD sample has smaller average crystallite size. The average crystallite size calculated by Scherrer equation of nanocrystals synthesized with EG was 24.9 nm, while the average crystallite size of nanocrystals produced with BD was 6.0 nm.

**Figure 2:**
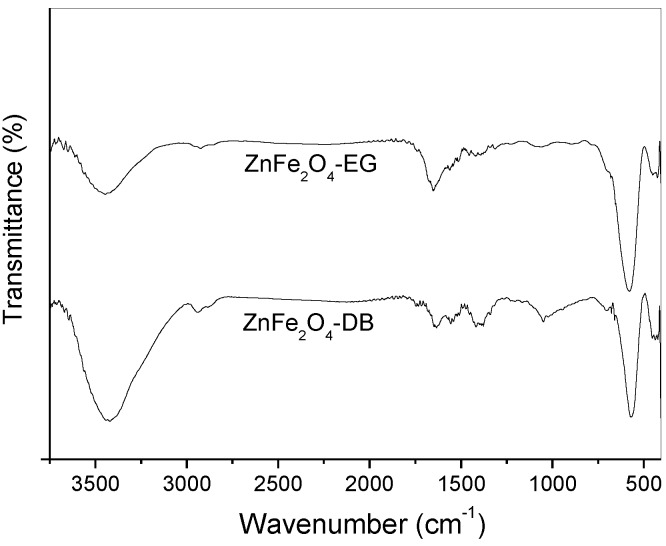
FTIR spectra of the samples prepared with different solvent diols.

FTIR spectra of the ferrite samples are presented in Figure 2. The bands at 3440 and 1640 cm^−1^ can be assigned to the stretching vibration mode of adsorbed water molecules on the surface of ferrite crystals [44,45]. However, the main bands that characterize the formation of spinel phase are located at 570 and 440 cm^−1^, which are associated with the vibrations of Zn-O and Fe-O bonds, respectively [27,44].

**Figure 3:**
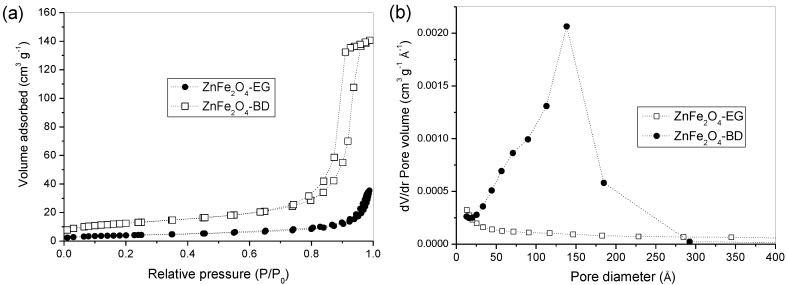
(**a**) N_2_ adsorption-desorption isotherms measured at 77 K; and (**b**) pore size distribution curves from the adsorption branches using the BJH method.

Figure 3 shows nitrogen adsorption-desorption isotherms (Figure 3a) of the obtained ZnFe_2_O_4_ samples and their corresponding pore size distribution curves (Figure 3b). As shown in Figure 3, the isotherms, as well as the pore size distribution curves of both samples, are significantly different. The nitrogen adsorption-desorption isotherms (Figure 3a) of the ZnFe_2_O_4_-BD sample are type IV with an H1 hysteresis loop according to the IUPAC classification, which indicates the predominance of mesoporous structure. While those of the ZnFe_2_O_4_-EG sample are of type III, indicating materials with predominantly microporous structure. The size pore distributions (Figure 3b) of the samples confirm the presence of mesoporous for the ZnFe_2_O_4_-BD sample and microporous for the ZnFe_2_O_4_-EG sample. Pore size distribution consisted of one wide peak centered at 150 Å (15 nm) for the ZnFe_2_O_4_-BD sample. This mesoporosity can be attributed to the interparticle pores due to the crystallites agglomeration. The specific surface area and total pore volume of the ZnFe_2_O_4_-BD sample were 44.6 m^2^·g^−1^ and 0.217 cm^3^·g^−1^ respectively, larger than those of the ZnFe_2_O_4_-EG sample, 14.6 m^2^·g^−1^ and 0.045 cm^3^·g^−1^ respectively. Different values of surface area and pore volume were found when different diols such as ethylene glycol, 1,2 propanediol, 2,3 butanediol and 2-methyl-2,4-pentanediol were used in the preparation of alumina-silica powders using the sol-gel method [46].

**Figure 4:**
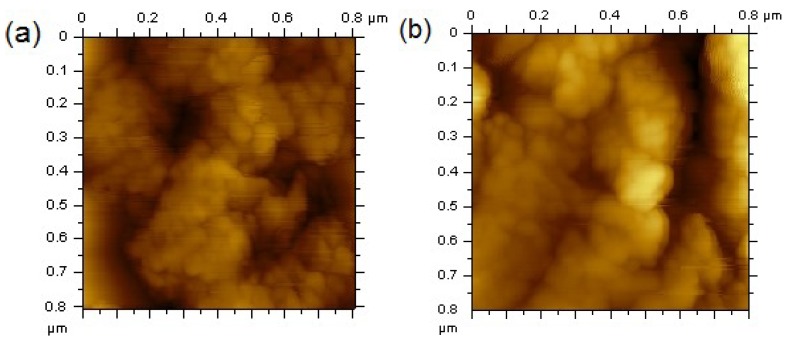
Atomic force microscopy (AFM) of (**a**) ZnFe_2_O_4_-BD and (**b**) ZnFe_2_O_4_-EG.

AFM images (Figure 4) show that the ZnFe_2_O_4_ samples prepared with EG and BD are formed by the agglomeration of small particles that are smaller than 50 nm, which are on the same order of magnitude of those calculated with the Scherrer equation in XRD analysis.

**Figure 5:**
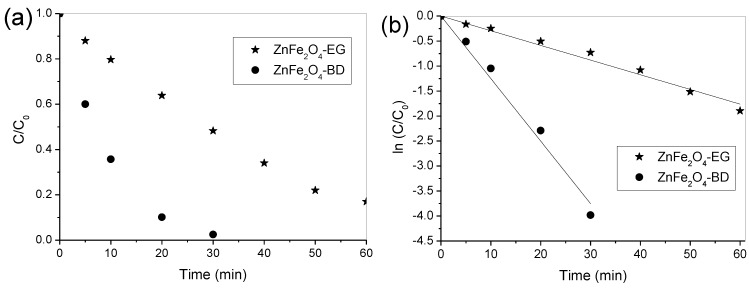
(**a**) Degradation profiles and (**b**) the variation of ln(*C*/*C*_0_) of Procion red dye over ZnFe_2_O_4_-EG and ZnFe_2_O_4_-DB. Reaction conditions: Initial H_2_O_2_ concentration = 0.04 mol·L^−^^1^, catalyst amount = 0.5 g, initial dye concentration = 50 mg·L^−1^ and initial pH = 3.0.

Preliminary experiments were performed in the conditions of photolysis (with presence of visible light irradiation only) and Fenton reaction (with catalyst and hydrogen peroxide in the absence of visible light irradiation), which negligible results (smaller 5% of dye degradation) were observed for both conditions. In addition, other experiments demonstrated that the dye degradation was negligible when using catalyst in the presence of visible light irradiation and without irradiation. Therefore, the photocatalytic activity of ZnFe_2_O_4_ powders only occurred in the simultaneous presence of visible light irradiation and hydrogen peroxide. Figure 5 depicts the photocatalytic activity of both ZnFe_2_O_4_ samples in the presence of visible light and hydrogen peroxide. ZnFe_2_O_4_-DB particles showed the highest photocatalytic activity for dye degradation, and complete removal occurred at 30 min of irradiation time, while the efficiency of ZnFe_2_O_4_-EG particles reached 85% of dye degradation at 60 min, as shown in Figure 5a. Thus, it is possible to note that the best catalytic performance occurs in the presence of ZnFe_2_O_4_-DB, and this may be associated with smaller crystallite size and, consequently, higher surface area. Figure 5b illustrates the reaction kinetics for the dye degradation using both catalysts prepared in this present work. The dye degradation followed the pseudo first-order kinetics [47,48] where the reaction rate constants (*k*) were obtained from slopes of the fit lines of ln(*C*/*C*_0_) *versus* reaction time. The reaction constants values were 29 × 10^−3^ min^−1^ (*R*^2^ = 0.99) and 125 × 10^−3^ min^−1^ (*R*^2^ = 0.99) for the ZnFe_2_O_4_-EG and ZnFe_2_O_4_-BD samples, respectively. Thus, ZnFe_2_O_4_-BD exhibited a rate that was about four times faster than that of ZnFe_2_O_4_-EG, which may associated with its higher surface area. Therefore, the results showed that the ZnFe_2_O_4_-BD sample displayed higher catalytic activity than that of the ZnFe_2_O_4_-EG sample under visible light irradiation. Due to its magnetic property [49], ZnFe_2_O_4_ spinel can be separated and recovered from aqueous solution through a magnetic field for further reutilization. The leaching of Fe ions in the solution was measured at 60 min irradiation for both catalysts. The concentrations of leached Fe were 4.2 and 4.5 mg·L^−1^ for the ZnFe_2_O_4_-BD and ZnFe_2_O_4_-EG catalysts, respectively, which are below the level established by the Brazilian environmental legislation (CONAMA) [50] for discharge in waste effluents, *i.e.*, 15 mg·L^−1^.

## 4. Conclusions

A solvothermal technique was used to produce ZnFe_2_O_4_ particles using two diol solvents. Results indicated that different physical properties may be found when different solvents are used for the synthesis of ZnFe_2_O_4_ particles. ZnFe_2_O_4_ particles were used as a heterogeneous photo-Fenton catalyst, exhibiting a good catalytic activity towards the degradation of Procion red dye in the presence of H_2_O_2_/visible light. Due to its greater surface area, ZnFe_2_O_4_-BD had a faster degradation rate compared to that of ZnFe_2_O_4_-EG. The photocatalytic degradation of Procion red dye from aqueous solution in the ZnFe_2_O_4_-visible irradiation-H_2_O_2_ system followed pseudo first-order kinetics. ZnFe_2_O_4_ catalysts prepared herein presented low iron leaching, and may be easily recovered and separated from aqueous solution with the aid of a magnetic field.
